# Accumulation of Nutrients and the Relation between Fruit, Grain, and Husk of Coffee Robusta Cultivated in Brazilian Amazon

**DOI:** 10.3390/plants12193476

**Published:** 2023-10-04

**Authors:** Raquel Schmidt, Cleidson Alves da Silva, Larícia Olária Emerick Silva, Marcelo Curitiba Espindula, Weverton Pereira Rodrigues, Henrique Duarte Vieira, Marcelo Antonio Tomaz, Fábio Luiz Partelli

**Affiliations:** 1 Center of Agricultural Sciences, Federal University of Espírito Santo, Alegre 29500-000, Espírito Santo, Braziltomazamarcelo@yahoo.com.br (M.A.T.); 2 Department of Agriculture, Federal University of Lavras, Lavras 37200-000, Minas Gerais, Brazil; 3 Rondônia Agroforestry Research Center, Brazilian Agricultural Research Corporation, Porto Velho 76815-800, Rondônia, Brazil; 4 Center of Agricultural, Natural and Literary Sciences, State University of the Tocantina Region of Maranhão, Estreito 65975-000, Maranhão, Brazil; weverton.rodrigues@uemasul.edu.br; 5 Agricultural Sciences and Technologies Center, State University of the North Fluminense Darcy Ribeiro, Campos dos Goytacazes 28013-602, Rio de Janeiro, Brazil; henrique@uenf.br; 6 Department of Agricultural and Biological Sciences, University Center of Northern Espírito Santo, Federal University of Espírito Santo, São Mateus 29932-900, Espírito Santo, Brazil

**Keywords:** *Coffea canephora*, genotypes, genetic breeding, nitrogen, plant nutrition, potassium

## Abstract

Coffee genotypes cultivated in the Amazonian region have been gaining increasing prominence in Brazilian plantations. This study aimed to quantify nutrient accumulation in the fruits, grains, and husks of Robusta coffee genotypes cultivated in the Brazilian Amazon and estimate genetic diversity. The experiment was conducted in Alta Floresta D’Oeste—Rondônia, Brazil. To assess nutrient accumulation, fresh fruits were collected. These were dried, processed, separated into grains and husks, and subjected to chemical analysis. Nutrient accumulation in fruits, grains, and husks, as well as the grain/husk ratio, underwent analysis of variance through the F-test (*p* < 0.01. For each evaluated trait, the experimental coefficient of 337 variation (CVe), genetic coefficient of variation (CVg), and genotypic determination coefficient (H^2^) were also estimated. Variability was observed among Robusta coffee genotypes, with VP06, AS4, and AS10 being the most dissimilar. LB080 had the lowest dry fruit weight and the lowest percentage of grains in relation to husks. ZD156 accumulated more K in the grains, while VP06 and AS10 were the genotypes that accumulated more nutrients in the husks. Nutrients N, K, Ca, and P are accumulated in larger quantities, necessitating the calibration of mineral fertilization dosages and distribution.

## 1. Introduction

*Coffea canephora* is an allogamous species and exhibits gametophytic self-incompatibility [1]. Gametophytic self-incompatibility is determined by the allele present in the pollen grain so that the pollen tube will only grow if the allele present in the pollen grain is not present in the genotype of the receiving plant [1]. This mechanism promotes genetic variability within the species, especially in native fields [2], seed-propagated plantations, and germplasm banks [3]. Therefore, seeds of *C. canephora* resulting from natural cross-pollination are generally genetically distinct. 

Among the methods used by *Coffea canephora* breeding programs to develop more productive, pest- and disease-resistant, drought and heat-tolerant cultivars, the selection of promising genotypes followed by vegetative propagation and compatibility testing is the most commonly used [4,5]. Typically, *C. canephora* cultivars consist of at least five genotypes (when compatibility is known) and at least nine (when compatibility is unknown) to achieve greater pollination efficiency [6]. However, it is rare for a single cultivar to encompass all desired characteristics.

Coffee has the ability to adapt to various types of soil but requires a substantial amount of nutrients [7]. The macronutrients nitrogen (N), phosphorus (P), potassium (K), and calcium (Ca) are the most crucial for coffee plants as they play roles in leaf expansion, floral bud formation, fruit filling, and overall plant development, among other functions [7]. However, the nutritional requirements may not always be the same for every coffee genotype and managing them in the same way can lead to imbalances in plant metabolism. The lack or unavailability of soil nutrients can affect it, including photosynthetic tissue loss, aerial part decrease and thus lower productive capacity [8], and greater susceptibility to pests and diseases [9]. Quantifying fruit, husk, and grain nutrient accumulation is important since results can foster soil fertilization practices, such as reusing husks in plantations to improve the soil and cycle nutrients [10], and optimize fertilization, restoring the nutrients removed by harvest and pruning.

Genotypes grown under Amazonian conditions, namely in Rondônia state, have increasingly gained space in Brazilian coffee fields. However, their excellence depends on their maintenance due to their high nutritional and water requirements [5]. Coffee growers specifically chose many such genotypes for their production and architecture [11]. Yield after processing is a pillar for the permanence of genotypes in the field since commercial plantations usually discard those with low ones [4]. Thus, information on nutrient accumulation in fruits, grains, and husks of different genetic materials is essential to genetically improve crops. Therefore, this study aimed to quantify the accumulation of nutrients in the fruits, grains, and husks, and to describe 16 *Coffea canephora* cv. Robusta genotypes cultivated in the Brazilian Amazon and estimated genetic diversity.

## 2. Results

### 2.1. Parameters and Genetic Diversity 

Most minerals showed experimental coefficients of variation (CVe) below 13%, except Fe (27.3%) and B (20.0%) (Table 1). We obtained genetic coefficient of variation (CVg) values above 10% for the accumulation of all nutrients except N (7.9%) and for grain/husk ratio (7.4%) (Table 1). Mn accumulation showed the highest genotypic coefficient of determination (H^2^) (95.7%), followed by sulfur (92.4%). Other minerals showed H^2^ above 69%—except Fe (37.4%) and B (54.6%). The grain/husk ratio also showed a high H^2^ value (92.4%).

### 2.2. Genotype Groupings and Genetic Contribution

**Figure 1:**
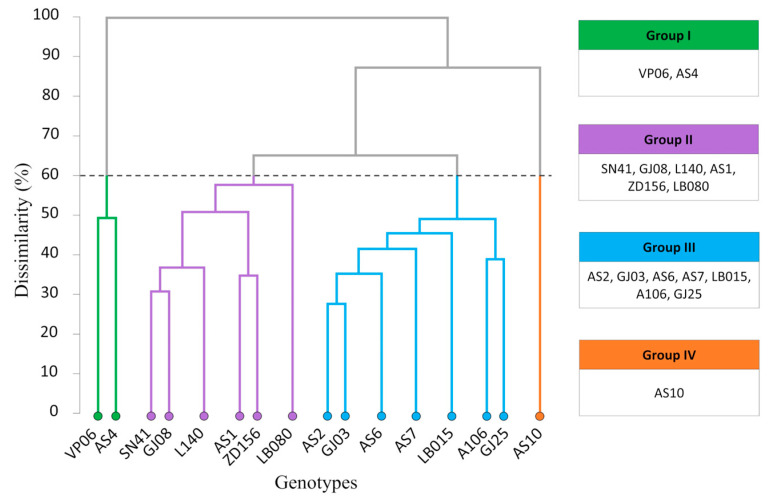
Dendrogram representing nutrient accumulation of 16 C. canephora cv. Robusta genotypes obtained by UPGMA via Euclidean distances. Cophenetic correlation: 0.77.

Considering fruit nutrient accumulation, we proposed grouping genotypes by UPGMA via their Euclidean distance. We arranged them into four groups, namely: Group I—VP06 and AS4; Group II—SN41, GJ08, L140, AS1, ZD156, and LB080; Group III (the most numerous)—AS2, GJ03, AS6, AS7, LB015, A106, and GJ25 and group IV—AS10 (Figure 1).

By Tocher’s method, we obtained four groups based on their Euclidean distances, which differed from UPGMA ones (Table 2). Group I included 11 genotypes (AS2, GJ03, AS6, GJ25, AS7, AS1, LB015, ZD156, SN41, GJ08, and L140); Group II, two (VP06, AS4); Group III; two, (A106, AS10) and Group IV, only one (LB080).

We used Singh’s method [12] to estimate the relative contribution of fruit nutrient accumulation and husk/grain ratios to genetic diversity (Table 3), which ranged from 23.4% to 0.1%. The accumulation of B (23.4%) and P (21.9%) were the traits that contributed the most to this study of genetic diversity.

### 2.3. Nutrient Accumulation in Fruits, Grains, and Husks

Significant differences among genotypes were observed for all traits except for Fe and B (Table 4). VP06, AS10, and AS4 fruits accumulated N in similarly greater quantities. AS10 accumulated the most P and Zn in its grains. All genotypes accumulated Fe and B in equal quantities. We considered VP06 as a highly nutritionally demanding genotype since it accumulated the most N, K, Ca, Mg, S, and Mn. GJ25 had K accumulation resembling VP06. On the other hand, L140, GJ08, LB080, and LB015 accumulated the least K and AS1, AS7, SN41, L140, GJ08, and LB080 fruits, the least P and Ca (Table 4). 

Significant differences among genotypes were observed for all traits except P, Fe, and B (Table 5). ZD156 accumulated more K, AS4, and GJ03, while VP06 accumulated more Ca, and VP06 and AS7 accumulated more S. On the other hand, SN41, GJ08, and GJ03 were the genotypes that accumulated the least Mg in the grains. Regarding the accumulation of Cu and Mn, the genotypes were divided into two groups (‘a’ and ‘b’). The genotypes with the highest means values for Cu were the same for Mn, except for AS7, ZD156 and LB015 (Table 5).

Regarding the accumulation of nutrients in the husk, only in the case of Fe were there no significant differences among genotypes (Table 6). VP06 accumulated the highest amounts of N, K, Ca, Mg, S, Fe, Mn, and B in the husk. AS10 was the second genotype that accumulated higher levels of P, Ca, Mg, S, Cu, Fe, and Zn. There was greater variation in the accumulation of P, K, S, and Mn (‘a’ to ‘e’) in the straw compared to fruits and grains (Table 6).

### 2.4. Fruit Weight and Husk/Grain Ratio

**Figure 2:**
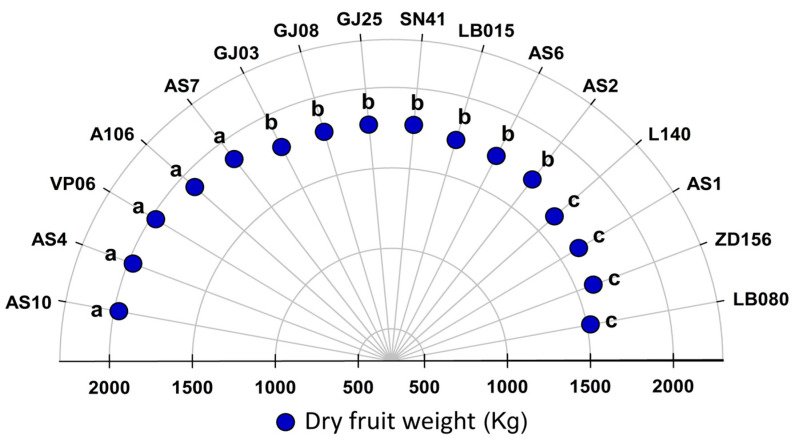
Dry fruit weight of 16 *C. canephora* cv. Robusta genotypes, considering one ton of grains. Grain drying was adjusted to a moisture content of 12% (wet basis). Means followed by the same letter belong to the same group according to Scott–Knot method at 5% probability.

We observed that AS10, AS4, VP06, A106, and AS7 showed the highest unprocessed dried fruit weight (without difference between them). L140, AS1, ZD156, and LB080 dry unprocessed samples showed the lowest weights. GJ03, GJ08, GJ25, SN41, LB015, AS6, and AS2 obtained similar dry fruit weight means (Figure 2).

**Figure 3:**
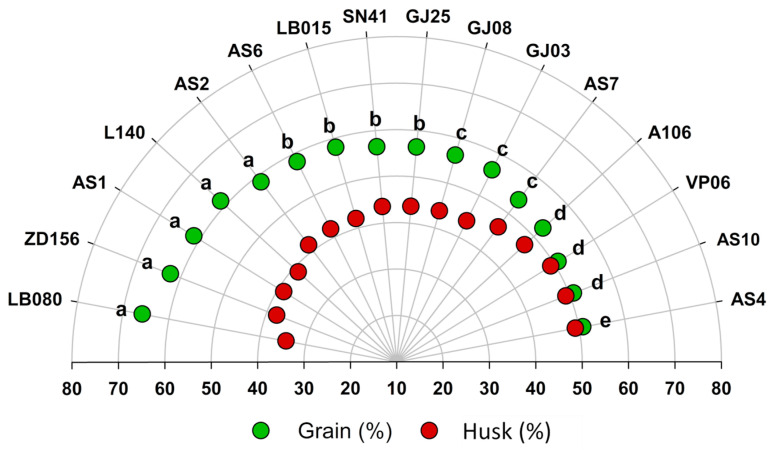
Grain/husk ratio in fruits of 16 *C. canephora* cv. Robusta genotypes. Grain and husk drying was adjusted to a moisture content of 12% (wet basis). Means followed by the same letter belong to the same group according to Scott–Knot method at 5% probability.

LB80, ZD156, AS1, L140, and AS2 constitute the group with the best grain/husk ratios, in which grains compose 60 to 70% of their fruit weight, followed by AS6, LB015, SN41, and GJ25, whose grains compose between 55 and 60% of their fruit weight. GJ08, GJ03, and AS7 showed around 50 to 55% of fruits to total weight; A106, VP06, and AS10, close to 50% and AS4, only 50% (the lowest yield). All genotypes obtained a ratio above 50% in the husk and grain conversion (Figure 3).

### 2.5. Correlation between Fruit Nutrient Accumulation and Husk to Grain Ratio

Correlation coefficients can range from −1 to +1. An absolute value of 1 indicates that the ordered data are perfectly linear. The closer the absolute value is to 1, the stronger the relationship between the variables. We observed 86 correlations among nutrient accumulations in grains, husks, and fruits (Table 7). Strong and positive correlations (above 0.8) were observed between GrA x FrA for P, between HuA x FrA for K, Ca, Mg, S, Cu, Fe, Mn, B, and between HuA x Hu% for S. Meanwhile, there was a strong negative correlation (above −0.8) between HuA x Gr% and S.

## 3. Discussion

### 3.1. Parameters and Genetic Diversity 

The estimation of genetic parameter experimental coefficient of variation (CVe), genetic coefficient of variation (CVg), and genotypic determination coefficient (H^2^) is frequently used in breeding programs [5,13], as they allow inferences about the genetic variability present in the population, in addition to the possibility of predicting genetic gains and success in the breeding program [13,14]. All evaluated traits presented CVe lower than 20%, except for the accumulation of Fe and B (Table 1). These results indicate that there was a low environmental influence on the expression of traits related to nutrient accumulation in coffee fruits, grains and husks. CVe determines how much the environment influences a particular phenotypic expression [14]. Based on the CVg values (Table 1), it was possible to observe that there were traits with greater genetic variability (e.g., manganese accumulation) than others (e.g., nitrogen accumulation). Manganese, sulfur, magnesium accumulation, and the grain/husk ratio showed the highest H^2^ values (Table 1). The higher and more accurate the H^2^, the more confident the breeder will be in predicting genetic gains [15]. In Conilon coffee genotypes, the concentration of Mn, Cu, N, P, Zn, and B in the grain also shows high H^2^ values [16,17].

### 3.2. Genotype Groupings and Genetic Contribution

Based on nutrient accumulation and the percentages of husk and grain, the UPGMA clustering of genotypes indicated four groups (Figure 1). The most dissimilar genotypes were VP06 and AS4 (Group I), and AS10 (Group IV). The genotypes within these two groups could be suggested for crossbreeding tests, as greater dissimilarity increases the chances of reproductive success. The Tocher method grouped the 16 genotypes slightly differently (Table 2). As a result, LB080 remained isolated in one group, while AS10 and A106 formed another. The high dissimilarity among genotypes significantly contributes to these methodological similarities [18]. C. canephora is an allogamous and self-incompatible species that naturally favors the uniqueness of each genotype, which may be visible in their phenotype. The more heterogeneous the study group, the more evident the dissimilarities between individuals [1].

In previous evaluations of nutrient concentration in different plant organs, six groups were formed for the same 16 genotypes of C. canephora cv. Robusta that we are evaluating here [5]. However, genotypes such as AS2, GJ03, and ZD156 remained in the same group, as in this experiment. Similarities among genotypical traits, such as the leaf, flower, fruit nutrient concentration, nutrient accumulation, fruit weight, and husk/grain ratio after processing, favor management during plant cycles, such as fertilization, irrigation, cultural tracts, and especially harvest, since frutos with similar cycles maturate in alike periods [19].

### 3.3. Fruit, Grain, and Husk Nutrient Accumulation

Coffee fruits have three morphological structures: pericarps (exocarp, mesocarp, endocarp), perisperms, and endosperms. After processing, grains are separated, originating “coffee husks” [4]. Evaluating fruit, husk, and grain nutrient accumulation is important, especially to correctly manage crop fertilization. Genotypes showing similar N, P, and K results facilitate nutritional management due to the common use of ready-formulated fertilizers such as 20-05-20 and 20-0-20 [6,20]. Regarding nutrients such as N (highly demanded and liable to several losses in soil-plant-atmosphere systems), researchers must know their absorption and accumulation dynamics in the various organs of plants since nursery [21,22].

In the 16 genotypes of C. canephora cv. Robusta evaluated, the accumulation of P was greater than Ca, corroborating the studies by [10]. In fruits and husks, the accumulation order we found resembled that observed in [10]. To produce one ton of Conilon coffee, the authors report the following descending order of husk (K > N > Ca > S > P > Mg > Fe > B > Mn > Zn > Cu), grain (N > K > P > Ca > S > Mg > Fe > B > Cu > Mn > Zn), and fruit (N > K > Ca > P > S > Mg > Fe > B > Cu > Mn > Zn) nutrient accumulation. In irrigated and non-irrigated coffee, fruits and leaves accumulated N, K, and Ca the most [19]. Thus, ensuring high yields requires a balanced fertilization management of these macronutrients. Genotypes such as ZD156 significantly accumulate K in their fruits. Crops that improperly correct this demand can produce low yields since K is one of the main nutrients responsible for grain filling and subsequent grain weight [23]. The lack of K, Ca, and S absorption and accumulation in plants, for example, can also affect their physiological processes and decrease dry aerial parts, reducing the viable number of plagiotropic branches and floral structures [24]. 

VP06 and AS10 show the highest nutrient accumulations in their husks. The literature has already reported the high nutrient accumulation in coffee husks, especially of N, P, K, Ca, and Mg [5,19] making nutrient cycling management important for plant development since the organic matter layer (0–20 cm) concentrates its roots and remove most nutrients [9,25].

### 3.4. Fruit Weight and Husk to Grain Ratio 

Dried fruit weight (Figure 2) differed among genotypes, indicating a genetic variability within the group. L140, AS1, SD156, and LB080 showed the best weights and averages of grain/husk ratio after processing. These traits raise the threshold of individuals in breeding programs. On the other hand, genotypes with low grain/husk ratios (AS4, AS10, VP06, A106, and AS7) may compromise yield during breeding trials or in commercial crops [4,26]. 

Fruit nutrient absorption, environmental conditions, and exact harvest period (at least 80% of ripe fruits) directly influence the accumulation of fresh mass [4]. The grain/husk ratio is a reference to establish whether a genotype is productive in a given environment. Its wide diversity in the field favors the selection of similar groups for harvests, thus facilitating post-harvest grain processing [18,26].

### 3.5. Correlation between Fruit Nutrient Accumulation and Husk to Grain Ratio

Moderate to strong positive correlations indicate how much certain traits influence the phenotype of each individual [27]. All nutrients obtained positive correlations for “husk x fruit accumulation,” especially Ca, Mg, Fe, and Mn (Table 7). Coffee crops highly require both macronutrients, especially during growth, flowering, filling, and yield [24,25]. Research should observe the nutritional demand of these nutrients with caution, especially regarding fertilization management before the critical sufficiency range, such as pre-flowering and fruit filling [28].

## 4. Materials and Methods

### 4.1. Experiment Installation and Area and Genotype Characterization

This study was conducted in the municipality of Alta Floresta D’Oeste—Rondônia, Brazil (Amazon region). It lies at latitude 12°08′51.86 S, longitude 62°04′95.03” W, 440 m.a.s.l and with minimum and maximum mean annual temperatures of 21 and 29.75 °C, and accumulated rainfall of 1965 mm. The local tropical climate has two distinct seasons, a dry one between June and October during the Amazonian summer and a rainy one between November and May during the Amazonian winter and the Köppen classification rates it as Aw [29]. The local soil is characterized as eutrophic Red Latosol [30]. Its chemical and physical characteristics are described in Table 8.

The experiment was conducted in April 2018 with 3.30 m between rows and 0.8 m between plants, totaling a density of 3.700 plants ha*^−^*^1^. The studied Amazonian Robusta genotypes were arranged in lines representing blocks. The plants were managed with two orthotropic branches (about 7.500 stems ha*^−^*^1^). The experiment was managed according to their needs aiming at the phytosanitary and nutritional management of the crop and they were drip irrigated to meet their water demands. In total, 400, 100, and 300 kg ha^1^ of N, P_2_O_5_, and K_2_O were administered to plants, respectively, and plotting was performed according to plant requirements and phenological stages.

The following *C. canephora* Pierre ex Froehner genotypes were used: A106, AS2, GJ25, VP06, AS1, AS7, SN41, AS6, ZD156, AS10, AS4, L140, GJ08, LB080, LB015, and GJ03. They were selected by coffee growers and nurserymen from Rondônia due to their productive potential.

### 4.2. Fruit Collection and Nutrient Analyses

The fruit collection was carried out from May to June 2020, when over 80% of the fruits were ripe. For nutrient accumulation, samples containing 2 L of fresh coffee were collected and then dried under direct sunlight. Subsequently, the samples were stored in properly labeled paper bags, placed in thermal boxes, and transported to the laboratory. To ensure uniform drying of the fruits, they were dried in a forced air circulation oven at 50 °C until they reached a constant weight. Once dried, the fruits were separated into grains and husks. The drying of the grains was adjusted to a moisture content of 12% (wet basis).

The samples underwent chemical analyses to determine the accumulation of minerals: nitrogen (N), phosphorus (P), potassium (K), calcium (Ca), magnesium (Mg), sulfur (S), iron (Fe), zinc (Zn), copper (Cu), manganese (Mn), and boron (B), following the methodology described by [31].

### 4.3. Grain and Husk Ratio

To assess the grain/husk ratio, 200 g fresh mass samples were collected and sundried. Grains and husks were then separated for processing. A completely randomized design was used in which 20 grains per genotype were thrice collected for processing, totaling 60 grains per genotype. After drying in a forced air circulation oven at 50 °C until constant weight, fruits were processed, and their grains and husks were separately weighed.

### 4.4. Statistical Analysis

The data underwent analysis of variance (ANOVA). After identifying significant differences among genotypes through ANOVA (*p* < 0.01), the means were grouped using the Scott–Knott method (*p* < 0.05). For each evaluated trait, the experimental coefficient of variation (CVe), genetic coefficient of variation (CVg), and genotypic determination coefficient (H^2^) were also estimated [32].

To assess genetic diversity, a Euclidean distance matrix was used to measure dissimilarities. Genotypes were grouped by the hierarchical unweighted pair group method with arithmetic mean (UPGMA) and Tocher’s method. The relative importance of nutrient accumulation and grain/husk ratio were also assessed to predict genetic diversity, as per [12]. Moreover, Spearman correlation coefficients for grain, husk, and fruit nutrient accumulation, grain and husk percentages in fruits, and fruit weight were obtained. Statistical analyses were performed via Genes [32].

## 5. Conclusions

The heterogeneity among the genotypic groups favors the application of our tests in breeding programs to characterize the differences and similarities among individuals. The genotypes were divided into four groups, with AS10 isolated in a distinct group. Genotype ZD156 exhibited the highest accumulation of K in the grains. VP06 and AS10 showed the highest nutrient accumulations in the husk. The genotype LB080 obtained the lowest dry fruit weight and the lowest percentage of grains in relation to husk, indicating that a larger quantity of dry fruits is necessary to obtain one ton of processed coffee. N, K, Ca, and P are accumulated in larger quantities, necessitating adjustments in dosages and timing of mineral fertilization. The incorporation of husks into the soil can aid in nutrient cycling and soil structure enhancement, thereby reducing the dependency on chemical fertilizers, and contributing to increasing the producer’s income and the sustainability of coffee farming.

## Figures and Tables

**Table 1:** Estimation of genetic parameters for nutrient accumulation and grain/husk ratios in 16 *C. canephora* cv. Robusta genotypes fruits, considering one ton of grains. N, nitrogen; P, phosphorus; K, potassium; Ca, calcium; Mg, magnesium; S, sulfur; Cu, copper; Fe: iron; Mn, manganese; Zn, zinc; B, boron and grain/husk ratio. CVe (%), experimental coefficient of variation; CVg (%), genetic coefficient of variation; H^2^ (%), genotypic coefficient of determination.

Variables	CVe (%)	CVg (%)	H^2^ (%)
N	9.2	7.9	69.2
P	9.2	13.8	86.9
K	10.2	16.5	88.6
Ca	12.7	18.0	85.8
Mn	8.1	14.5	90.6
S	8.5	17.1	92.4
Cu	11.5	15.9	85.2
F	27.3	12.1	37.4
Mn	7.3	19.9	95.7
Zn	12.2	17.3	85.7
B	20.0	12.7	54.6
Grain/husk	3.7	7.4	92.4

**Table 2:** Tocher’s clusters of 16 C. canephora cv. Robusta genotypes based on Euclidean distances, considering fruit nutrient accumulation and husk/grain ratios.

Groups	Genotypes
I	AS2, GJ03, AS6, GJ25, AS7, AS1, LB015, ZD156, SN41, GJ08, L140
II	VP06, AS4
III	A106, AS10
IV	LB080

**Table 3:** Relative contribution of fruit nutrient accumulation and grain/husk ratio for the genetic diversity of 16 C. canephora cv. Robusta genotypes, according to Singh’s method (1981). N, nitrogen; P, phosphorus; K, potassium; Ca, calcium; Mg, magnesium; S, sulfur; Cu, copper; Mn, manganese; Zn, zinc; B, boron and grain/husk ratio.

Variables	S.j	Value (%)	Accumulated Value (%)
N	1584.9	6.3	95.6
P	43.5	0.2	99.9
K	5472.4	21.9	45.3
Ca	242.1	0.9	99.3
Mg	25.9	0.1	100.0
S	105.6	0.4	99.7
Cu	2417.0	9.7	89.2
Mn	3889.5	15.6	79.5
Zn	693.4	2.8	98.3
B	5843.6	23.4	23.4
Grain/husk	4665.7	18.7	63.9

S.j (Singh, 1981) [12].

**Table 4:** Grouping of means of accumulation of nutrients in the fruits (grain + husk) of 16 *C. canephora* cv. Robusta genotypes, considering one ton of grains. Fruits drying was adjusted to a moisture content of 12% (wet basis). N, nitrogen; P, phosphorus; K, potassium; Ca, calcium; Mg, magnesium; S, sulfur; Cu, copper; Fe: iron; Mn, manganese; Zn, zinc; B, boron.

			Nutrients								
Genotypes	N	P	K	Ca	Mg	S	Cu	Fe	Mn	Zn	B
	—————— kg.ton^−1^ ——————						————— g.ton^−1^ —————				
A106	26.5 b	2.9 b	28.7 b	5.3 b	2.3 b	4.1 b	26.4 a	50.8 a	19.4 d	11.0 b	31.2 a
AS2	23.6 b	2.7 c	27.3 b	5.5 b	2.2 b	3.9 b	19.6 b	44.6 a	19.4 d	8.9 c	29.5 a
GJ25	24.3 b	3.1 b	33.1 a	5.2 b	2.0 c	3.8 b	21.9 b	43.2 a	21.2 c	9.7 c	31.7 a
VP06	32.9 a	2.5 c	37.6 a	6.8 a	2.8 a	5.2 a	19.7 b	40.9 a	29.3 a	10.8 b	36.8 a
AS1	27.1 b	2.5 c	27.1 b	4.4 c	2.1 b	3.3 c	16.2 c	51.8 a	20.5 c	7.7 c	27.1 a
AS7	26.8 b	2.6 c	27.7 b	4.9 c	2.2 b	4.4 b	17.9 c	39.5 a	18.8 d	8.9 c	33.3 a
SN41	24.9 b	2.7 c	27.9 b	4.1 c	1.5 d	3.9 b	16.9 c	39.4 a	14.4 e	7.6 c	28.6 a
AS6	28.1 b	2.9 b	28.1 b	5.7 b	2.0 c	3.7 b	18.4 c	48.0 a	21.5 c	7.1 c	23.1 a
ZD156	27.5 b	3.2 b	30.3 b	4.0 c	2.3 b	3.1 c	18.6 c	30.3 a	17.2 d	9.1 c	23.8 a
AS10	29.1 a	4.1 a	30.8 b	5.8 b	2.3 b	3.9 b	21.1 b	37.0 a	19.3 d	13.1 a	24.8 a
AS4	30.6 a	3.2 b	28.7 b	6.8 a	2.6 a	4.4 b	19.8 b	35.9 a	25.9 b	8.8 c	39.3 a
L140	23.2 b	2.3 c	21.5 c	4.0 c	1.8 c	3.2 c	15.2 c	29.8 a	15.0 e	8.2 c	29.2 a
GJ08	26.2 b	2.6 c	23.0 c	4.1 c	1.7 d	3.1 c	12.9 c	36.5 a	16.2 e	7.7 c	26.8 a
LB080	25.8 b	2.4 c	18.1 c	3.7 c	1.7 d	2.3 d	14.7 c	26.8 a	14.4 e	6.9 c	20.4 a
LB015	24.9 b	2.8 b	20.9 c	5.7 b	2.3 b	3.2 c	17.1 c	30.0 a	21.2 c	11.3 b	27.4 a
GJ03	26.1 b	2.9 b	25.8 b	5.9 b	2.1 b	3.7 b	17.4 c	32.3 a	22.4 c	8.6 c	27.1 a
	Variance analysis										
Genotypes	3.3 **	7.8 **	8.8 **	7.0 **	10.9 **	13.2 **	6.7 **	1.6 ^ns^	23.5 **	7.0 **	2.2 ^ns^
CV (%)	9.2	9.2	10.2	12.7	8.0	8.5	11.5	27.3	7.3	12.2	20.0
Mean	26.7	2.8	27.3	5.1	2.2	3.7	18.4	38.6	19.8	9.1	28.7

Means followed by the same letter in the columns belong to the same group by Scott–Knott method at 5% probability. ‘**’ corresponded to the significance of *p* < 0.01. ‘ns’ not significant were indicated.

**Table 5:** Grouping of means of accumulation of nutrients in the grains of 16 of C. canephora cv. Robusta genotypes, considering one ton of grains. Grains drying was adjusted to a moisture content of 12% (wet basis). N, nitrogen; P, phosphorus; K, potassium; Ca, calcium; Mg, magnesium; S, sulfur; Cu, copper; Fe: iron; Mn, manganese; Zn, zinc; B, boron.

	Nutrients										
Genotypes	N	P	K	Ca	Mg	S	Cu	Fe	Mn	Zn	B
	—————— kg.ton^−1^ ——————						————— g.ton^−1^ —————				
A106	19.2 b	1.5 a	12.2 b	1.9 c	1.3 a	1.5 b	13.3 a	19.4 a	11.8 a	5.8 a	11.3 a
AS2	19.2 b	1.7 a	12.9 b	2.2 b	1.3 a	1.7 b	11.8 a	15.7 a	12.1 a	4.4 b	13.6 a
GJ25	18.2 b	2.0 a	14.3 b	2.3 b	1.3 a	1.6 b	12.6 a	17.1 a	12.8 a	5.3 b	15.1 a
VP06	20.3 a	1.8 a	12.8 b	2.6 a	1.6 a	2.1 a	11.2 a	17.4 a	13.4 a	6.7 a	11.7 a
AS1	21.5 a	1.8 a	13.5 b	2.2 b	1.4 a	1.6 b	11.5 a	20.6 a	12.9 a	5.2 b	12.9 a
AS7	19.9 b	1.7 a	12.3 b	1.9 c	1.3 a	2.0 a	10.4 a	16.9 a	11.1 b	4.8 b	16.5 a
SN41	18.4 b	1.7 a	11.8 b	1.7 c	0.9 b	1.4 b	9.3 b	18.4 a	8.9 b	3.3 b	11.9 a
AS6	22.2 a	1.8 a	11.9 b	2.1 b	1.3 a	1.5 b	9.7 b	19.4 a	11.1 b	4.4 b	12.9 a
ZD156	21.4 a	2.0 a	17.6 a	1.9 c	1.2 a	1.2 c	11.6 a	18.9 a	10.5 b	4.4 b	13.9 a
AS10	20.4 a	2.2 a	10.4 c	1.8 c	1.3 a	1.1 c	9.6 b	19.7 a	11.1 b	4.9 b	16.7 a
AS4	21.1 a	1.8 a	12.6 b	2.4 a	1.3 a	1.4 b	10.7 a	18.2 a	12.5 a	4.4 b	11.6 a
L140	18.6 b	1.5 a	9.9 c	1.8 c	1.2 a	1.4 c	8.6 b	16.8 a	9.7 b	6.2 a	14.5 a
GJ08	21.1 a	1.7 a	10.9 c	1.6 c	1.0 b	1.1 c	6.5 b	20.6 a	9.7 b	3.9 b	16.8 a
LB080	21.9 a	1.7 a	10.1 c	1.9 c	1.3 a	1.7 b	9.2 b	17.9 a	10.3 b	4.8 b	11.8 a
LB015	18.9 b	1.7 a	10.9 c	2.2 b	1.1 b	1.17 c	8.6 b	18.1 a	12.2 a	6.45 a	11.3 a
GJ03	19.9 b	1.8 a	13.2 b	2.4 a	1.4 a	1.36 c	10.6 a	19.4 a	12.2 a	5.13 b	10.7 a
	Variance analysis										
Genotypes	2.51 *	1.95 ^ns^	5.73 **	9.29 **	5.41 **	6.29 **	4.03 **	1.17 ^ns^	4.98 **	4.00 **	1.46 ^ns^
CV (%)	6.95	12.6	11.1	7.47	9.33	14.1	14.4	12.1	9.02	15.6	22.4
Mean	20.2	1.79	12.3	2.05	1.28	1.52	10.3	18.4	11.4	5.02	13.4

Means followed by the same letter in the columns belong to the same group by Skott–Knott method at 5% probability. ‘*’ and ‘**’corresponded to the significance of *p* < 0.05, 0.01, respectively. ‘ns’ not significant were indicated.

**Table 6:** Grouping of means of accumulation of nutrients in the husks of 16 of C. canephora cv. Robusta genotypes, considering one ton of grains. Husks drying was adjusted to a moisture content of 12% (wet basis). N, nitrogen; P, phosphorus; K, potassium; Ca, calcium; Mg, magnesium; S, sulfur; Cu, copper; Fe: iron; Mn, manganese; Zn, zinc; B, boron.

Genotypes	N	P	K	Ca	Mg	S	Cu	Fe	Mn	Zn	B
	—————— kg.ton^−1^ ——————						————— g.ton^−1^ —————				
A106	7.3 c	1.42 b	16.5 c	3.4 a	1.1 a	2.61 b	13.0 a	31.4 a	7.7 d	5.1 b	19.9 a
AS2	4.4 c	1.07 c	14.5 c	3.4 a	0.9 b	2.23 c	7.84 b	28.9 a	7.3 d	4.5 b	15.9 b
GJ25	6.1 c	1.10 c	18.8 b	2.9 b	0.7 b	2.2 c	9.3 b	26.1 a	8.3 d	4.8 b	16.7 b
VP06	12.6 a	0.80 d	24.8 a	4.3 a	1.2 a	3.1 a	8.4 b	23.5 a	15.9 a	4.1 b	25.1 a
AS1	5.6 c	0.7 d	13.7 c	2.2 b	0.8 b	1.7 d	4.7 b	31.2 a	7.5 d	2.5 c	14.2 b
AS7	6.9 c	0.9 c	15.3 c	3.0 b	0.9 a	2.4 b	7.5 b	22.5 a	7.7 d	4.1 b	16.8 b
SN41	6.6 c	1.0 c	16.2 c	2.4 b	0.6 b	2.2 c	7.6 b	20.9 a	5.5 e	4.3 b	16.6 b
AS6	5.9 c	1.1 c	16.1 c	3.7 a	0.7 b	2.2 c	8.7 b	28.6 a	10.4 c	2.7 c	10.2 c
ZD156	6.1 c	1.1 c	12.6 d	2.1 b	1.1 a	1.9 d	7.0 b	11.5 a	6.8 e	4.7 b	9.9 c
AS10	8.8 b	1.9 a	20.4 b	4.1 a	1.1 a	2.9 a	11.5 a	17.4 a	8.2 d	8.2 a	8.2 c
AS4	9.5 b	1.4 b	16.1 c	4.5 a	1.3 a	2.7 b	9.1 b	17.6 a	13.4 b	4.4 b	27.8 a
L140	4.3 c	0.8 d	11.7 d	2.2 b	0.7 b	1.8 d	6.7 b	13.1 a	5.3 e	2.2 c	14.3 b
GJ08	5.1 c	0.9 c	12.1 d	2.6 b	0.7 b	2.1 c	6.5 b	15.9 a	6.5 e	3.8 b	9.9 c
LB080	3.9 c	0.7 d	8.1 d	1.9 b	0.5 b	0.7 e	5.5 b	8.9 a	4.2 e	2.1 c	8.7 c
LB015	5.9 c	1.1 c	10.0 d	3.6 a	1.2 a	2.1 c	8.6 b	12.0 a	8.9 c	4.9 b	16.1 b
GJ03	6.2 c	1.1 c	12.7 d	3.6 a	0.8 b	2.4 b	6.9 b	12.9 a	10.2 c	3.5 b	16.4 b
	Variance analysis										
Genotypes	4.28 **	14.9 **	7.1 **	5.4 **	8.9 **	18.3 **	3.8 **	1.6 ^ns^	20.5 **	9.8 **	5.2 **
CV (%)	28.0	12.5	17.7	19.7	15.9	9.9	23.4	51.9	13.6	19.7	27.4
Mean	6.57	1.08	14.9	3.12	0.9	2.2	8.1	20.2	8.4	4.1	15.4

Means followed by the same letter in the columns belong to the same group by Skott–Knott method at 5% probability. ‘**’ corresponded to the significance of *p* < 0.01. ‘ns’ not significant were indicated.

**Table 7:** Spearman correlation coefficients for fruit, grain, husk nutrient accumulation, grain/husk ratio, and fruit weight in 16 of *C. canephora* cv. Robusta genotypes. GrA, Grain accumulation; HuA, Husk accumulation; FrA, Fruit accumulation; Gr%, percentage of grain; Hu%, percentage of husk; FrW, fruit weight. The correlations between two parameters were represented by ‘x’ for each nutrient.

Variables	Nutrients										
	N	P	K	Ca	Mg	S	Cu	Fe	Mn	Zn	B
GrA x HuA	0.00	0.26	0.21	0.47	0.18	0.12	0.14	−0.13	0.55	0.03	−0.26
GrA x FrA	0.62 **	0.80 **	0.55 **	0.64 **	0.54 **	0.59 **	0.66 **	0.15	0.76 **	0.57 **	0.17
GrA x Gr%	0.18	−0.09	−0.03	−0.19	−0.12	−0.14	−0.05	0.07	−0.23	−0.15	−0.01
GrA x Hu%	−0.18	0.09	0.03	0.19	0.12	0.14	0.05	−0.07	0.23	0.15	0.01
GrA x FrW	−0.18	0.09	0.03	0.19	0.12	0.14	0.05	−0.07	0.23	0.15	0.01
HuA x FrA	0.76 **	0.74 **	0.89 **	0.96 **	0.90 **	0.83 **	0.80 **	0.93 **	0.94 **	0.78 **	0.87 **
HuA x Gr%	−0.65 **	−0.58 **	−0.68 **	−0.72 **	−0.55 **	−0.92 **	−0.68 **	−0.45 **	−0.58 **	−0.61 **	−0.47 **
HuA x Hu%	0.65 **	0.58 **	0.68 **	0.72 **	0.55 **	0.92 **	0.68 **	0.45 **	0.58 **	0.61 **	0.47 **
HuA x FrW	0.65 **	0.58 **	0.68 **	0.72 **	0.55 **	0.92 **	0.68 **	0.45 **	0.58 **	0.61 **	0.47 **
FrA x Gr%	−0.38 **	−0.39 **	−0.52 **	−0.66 **	−0.52 **	−0.79 **	−0.55 **	−0.36 *	−0.47 **	−0.58 **	−0.54 **
FrA x Hu%	0.38 **	0.39 **	0.52 **	0.66 **	0.52 **	0.79 **	0.55 **	0.36 *	0.47 **	0.58 **	0.54 **
FrA x FrW	0.38 **	0.39 **	0.52 **	0.66 **	0.52 **	0.79 **	0.55 **	0.36 *	0.47 **	0.58 **	0.54 **
Gr% x Hu%	−1.00										
Gr% x FrW	−1.00										
Hu% x FrW	1.00										

* and ** corresponded to the significance of *p* < 0.05, 0.01, respectively.

**Table 8:** Granulometric and chemical characteristics of six soil depths of a eutrophic Red Latosol in an area cultivated with irrigated coffee (*C. canephora*) in Alta Floresta D’Oeste, Rondônia, Brazil. P, phosphorus; K, potassium; S, sulfur; Ca, calcium; Mg, magnesium; Al, aluminum; H + Al, Potential soil acidity; SOM: soil organic matter; Fe: iron; Zn, zinc; Cu, copper; Mn, manganese; B, boron; Na, sodium; CEC: cation exchange capability.

Particle Size Distribution	Soil Depth (cm)					
	0–10	10–20	20–30	30–40	40–50	50–60
Total sand (g kg^−1^)	172	180	180	174	174	198
Silt (g kg^−1^)	428	400	440	406	386	342
Clay (g kg^−1^)	400	420	380	420	440	460
	silty clay		silty clay loam	silty clay	clay	
**Soil Chemical Properties**	**Soil Depth (cm)**					
	**0–10**	**10–20**	**20–30**	**30–40**	**40–50**	**50–60**
P (mg kg^−1^)	3	11	5	3	2	14
K (mg kg^−1^)	44	87	72	60	48	13
S (mg kg^−1^)	5	10	7	7	4	8
Ca (cmol kg^−1^)	4.4	4.7	4.8	4.4	4.5	4.8
Mg (cmol kg^−1^)	0.7	0.8	0.7	0.7	0.7	0.9
Al (cmol kg^−1^)	0.0	0.3	0.1	0.0	0.0	0.5
H + Al (cmol dm^−3^)	3.1	4.2	3.6	3.3	3.3	5.0
pH-H_2_O	5.8	5.3	5.6	5.8	5.9	5.5
SOM (g kg^−1^)	2.1	2.5	2.5	2.1	2.4	3.1
Fe (mg kg^−1^)	111	80	99	96	92	78
Zn (mg kg^−1^)	1.2	9.9	1.7	1.8	1.5	8.8
Cu (mg kg^−1^)	2.4	2.8	2.8	2.8	2.4	4.7
Mn (mg kg^−1^)	184	208	196	207	168	287
B (mg kg^−1^)	0.25	0.58	0.62	0.83	0.51	0.71
Na (mg kg^−1^)	6.0	7.0	5.0	6.0	5.0	9.0
CEC (cmol kg^−1^)	8.34	9.95	9.31	8.58	8.64	11.07

## Data Availability

Publicly available datasets were analyzed in this study. The authors can provide the experimental data for all interested researchers.
